# A Novel Microfluidic Device for the Neutrophil Functional Phenotype Analysis: Effects of Glucose and Its Derivatives AGEs

**DOI:** 10.3390/mi12080944

**Published:** 2021-08-11

**Authors:** Ke Yang, Xiao Yang, Chaoru Gao, Changyi Hua, Chenggang Hong, Ling Zhu

**Affiliations:** 1 Anhui Institute of Optics and Fine Mechanics, Hefei Institutes of Physical Science, Chinese Academy of Sciences, Hefei 230031, China; xuehualang@aiofm.ac.cn; 2 School of Biomedical Engineering, Anhui Medical University, Hefei 230032, China; xiaoyang9806@outlook.com (X.Y.); gcr15805601879@outlook.com (C.G.); 3 Hefei Zhongke Yikangda Biomedical Co., Ltd., Hefei 230088, China; 13665603893@163.com

**Keywords:** neutrophil chemotaxis, diabetes mellitus, microfluidic device, glucose, AGEs

## Abstract

Neutrophil dysfunction is closely related to the pathophysiology of patients with diabetes mellitus, but existing immunoassays are difficult to implement in clinical applications, and neutrophil’s chemotaxis as a functional biomarker for diabetes mellitus prognostic remains largely unexplored. Herein, a novel microfluidic device consisted of four independent test units with four cell docking structures was developed to study the neutrophil chemotaxis, which allowed multiple cell migration observations under a single field of view (FOV) and guaranteed more reliable results. In vitro studies, the chemotaxis of healthy neutrophils to *N*-Formyl-Met-Leu-Phe (fMLP) gradient (0, 10, 100, and 1000 nM) was concentration-dependent. The distinct promotion or suppression in the chemotaxis of metformin or pravastatin pretreated cells were observed after exposure to 100 nM fMLP gradient, indicating the feasibility and efficiency of this novel microfluidic device for clinically relevant evaluation of neutrophil functional phenotype. Further, the chemotaxis of neutrophils pretreated with 25, 50, or 70 mM of glucose was quantitatively lower than that of the control groups (i.e., 5 mM normal serum level). Neutrophils exposed to highly concentrated advanced glycation end products (AGEs) (0.2, 0.5, or 1.0 μM; 0.13 μM normal serum AGEs level), a product of prolonged hyperglycemia, showed that the higher the AGEs concentration was, the weaker the migration speed became. Specifically, neutrophils exposed to high concentrations of glucose or AGEs also showed a stronger drifting along with the flow, further demonstrating the change of neutrophil chemotaxis. Interestingly, adding the *N*-benzyl-4-chloro-*N*-cyclohexylbenzamide (FPS-ZM1) (i.e., high-affinity RAGE inhibitor) into the migration medium with AGEs could hinder the binding between AGEs and AGE receptor (RAGE) located on the neutrophil, thereby keeping the normal chemotaxis of neutrophils than the ones incubated with AGEs alone. These results revealed the negative effects of high concentrations of glucose and AGEs on the neutrophil chemotaxis, suggesting that patients with diabetes should manage serum AGEs and also pay attention to blood glucose indexes. Overall, this novel microfluidic device could significantly characterize the chemotaxis of neutrophils and have the potential to be further improved into a tool for risk stratification of diabetes mellitus.

## 1. Introduction

Diabetes mellitus is a prototypic dysmetabolic syndrome characterized by hyperglycemia, accompanied by functional and structural changes of multiple tissues, and eventually leads to the damage and failure of multiple organs [1,2]. Neutrophils are the most abundant leukocytes, which play a crucial role in diabetes mellitus pathogenesis and its related vascular complications [3,4]. In patients with diabetes mellitus, clinical researchers have found defective neutrophil functions, increased leukocyte counts, and cell morphological changes [5,6,7]. Developing a comprehensive and robust neutrophil phenotyping strategy is crucial for clarifying the pathogenesis in diabetes and related metabolic diseases.

Hyperglycemia can affect the immune response in different ways, including reducing the chemotaxis of immune cells and phagocytosis [6,8,9,10]. In addition, hyperglycemia has been demonstrated to cause changes in neutrophil adhesion in vitro, which can impede neutrophil transmigration from blood vessels toward infected tissues in vivo [11,12]. Moreover, long-term hyperglycemia will also accelerate the production of advanced glycation end products (AGEs), which have been proved to be closely related to the occurrence and development of immune dysfunction and vascular complications (atherosclerosis, etc.) in diabetic patients [13,14,15]. AGEs can also cause neutrophils to accelerate the production of reactive oxygen species (ROS), and excessive ROS is widely considered an important mediator for the development of diabetes-related vascular diseases [16]. In addition, AGEs are found to interact with receptors for AGE (RAGE) in vitro on cultured neutrophils, leading to decreased transendothelial cell migration and bactericidal capacity [17,18,19]. To sum up, previous studies stress the significance of further exploring the neutrophil function mediated by glucose and AGEs and associated health problems.

Microfluidic devices have been widely used to study cell migration and chemotaxis during the past 20 years due to the superiority of miniaturization and micro-environmental control [20,21,22,23]. In addition, microfluidic devices have been developed and improved to be diagnostic tools for neutrophil migration-related diseases [24,25,26]. More specifically, a few research groups [27,28,29,30,31,32,33] have promoted the development of functional phenotypes for diseases related to neutrophils’ migration on microfluidic devices. A previous study from our group reported the chemotaxis of human blood neutrophils mediated by Fibroblast growth factor 23 on a microfluidic device [34]. Furthermore, our group also demonstrated the advantage and feasibility of integrating the microfluidic device into a smartphone for studying the chemotaxis of neutrophils from normal volunteers and chronic obstructive pulmonary disease (COPD) patients in hospital testing [35]. The previous advanced results laid a solid foundation for functional phenotype research of cell migration and chemotaxis based on the microfluidic device. However, most microfluidic devices are only suitable for monitoring the cell migration in a single microchannel under one field of view (FOV) for each experiment, limiting the applications for parallel and comparable study on neutrophil chemotaxis under different experimental conditions [22,36,37]. Although parallelly arranging multiple microfluidic devices can improve the detection throughput, this method still cannot observe multiple cell migration experiments simultaneously under a single FOV [20,32]. It is necessary to move the microscope or microfluidic device for observing multiple channels, which is complicated and will increase time cost and labor intensity. Moreover, on-chip chemotaxis phenotype analysis of neutrophils exposure to high concentrations of glucose and AGEs remains largely unexplored, although they are of vital importance for diabetes mellitus pathogenesis.

Herein, a novel and high throughput version of a microfluidic device consisted of four independent channels (denoted as F4-Chip) was developed, which can perform multiple chemotaxis assays each time. Each experimental unit incorporated a cell docking structure to calibrate the initial neutrophil position and partially mimic the neutrophil transmigrating across endothelial cells, which guarantees more reliable chemotaxis results. The performance of the F4-Chip for the assessment of neutrophil functions was evaluated using the healthy neutrophils directly exposed to the fMLP chemoattractant gradient or pre-incubated with the diabetes treatment drugs (metformin and pravastatin) and then exposed to the fMLP. Then, the chemotaxis response of neutrophils incubated in high concentrations of glucose and AGEs was studied thoroughly. Distinct neutrophil chemotaxis suppression was observed in vitro inflamed neutrophils (high glucose). Intriguingly, neutrophils exposed to a high concentration of AGEs in vitro led to overall suppression in the chemotaxis behavior, while it could be recovered after the addition of *N*-benzyl-4-chloro-*N*-cyclohexylbenzamide (FPS-ZM1, RAGE blocker). Collectively, the advantages of the F4-Chip for inflammatory cytokines-related cell functional phenotype research were demonstrated. This novel F4-Chip was efficient in analyzing the phenotype of neutrophil migration and chemotaxis, which can be upgraded into a more advanced cell chemotaxis research tool (e.g., matching rapid neutrophils separation unit) for risk stratification in diabetes mellitus.

## 2. Materials and Methods

### 2.1. F4-Chip Design and Fabrication

**Figure 1:**
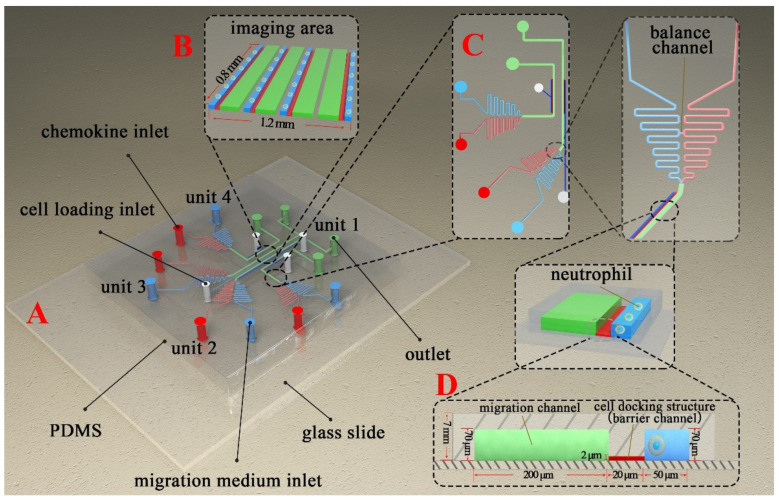
Schematic diagram of the F4-Chip used for cell chemotaxis research. (**A**) The isometric view of F4-Chip. (**B**) Enlarged view of the imaging area. (**C**) Enlarged view of unit 3 and unit 4. Each unit has its specific cell loading inlet, reagent inlet, and outlet. In addition, each unit has a balance channel to balance the pressure between the chemokine inlet and migration medium inlet. (**D**) The enlarged cell docking structure.

Photolithography and soft-lithography technology were used for fabricating the F4 chip [38]. The prototype of the F4-chip was designed using the Solidworks software (Version 2018, Dassault Systemes, Boston, MA, USA) and then manufactured in the Micro/Nano Research and Manufacturing Center, University of Science and Technology of China. Two-layer photolithography technology was used for creating the master mold. The geometry of the microchannel on the silicon wafer was created by patterning two layers of SU-8 photoresist with different thicknesses through double exposures. The first layer (2 μm thick) was used to create the docking structures. The second layer (70 μm thick) was used to create the main microchannels. Polydimethylsiloxane (PDMS) (Sylgard 184, Dow Corning) was poured on the master mold to create the main body of the F4-chip by soft-lithography technology. The PDMS replica with the inlets and outlets punched was then connected to the glass slide (Haide Biotechnology Co., LTD., Beijing, China, 71862-01, 75 mm × 51 mm × 1.2 mm) by air plasma treatment (PDC-MG, Chengdu Mingheng Technology Development Co., Ltd., Chengdu, China). The F4-chip was then coated with deionized water and placed in a biological safety cabinet for UV sterilization before being stored in a refrigerator at −4 °C for later use. Before the experiment, the F4-chip was first coated with the fibronectin (F2006, 1 mg/mL, SIGMA-Aldrich) for 60 min. and then incubated using the migration medium (0.4% BSA diluted by RPMI-1640) for 60 min. BSA (Order NO. A600332) and RPMI-1640 (Order NO. E600028) were purchased from Shanghai Sangon Biological Engineering Co., Ltd. A new F4 chip was used for every new experiment. The detailed structure of the F4 chip is shown in Figure 1. The detailed preparation progress of the F4 chip is shown in Figure S1.

### 2.2. Neutrophil Isolation

The chemotaxis experiments were all carried out according to the rules and regulations formulated by the Hefei Institute of Physical Science, Chinese Academy of Sciences. The Ethics Committee at the Hefei Institute of Physical Science was approved to obtain fresh venous blood from the healthy volunteers (Y-2018-21). A commercial neutrophil isolation kit was used to purify the cells (17957, STEMCELL Technologies, Inc., Toronto, Canada). The kit was coupled with a commercial magnet (18001, EasySep Direct, STEMCELL Technologies, Inc.).

### 2.3. Neutrophil Pretreatment

The detailed setting of the neutrophil’s incubation and pretreatment for the following experiments are provided in Figure S2.

Control cells incubation. The purified, healthy neutrophils were suspended in the migration medium and kept in an incubator (Shanghai Boxun Instrument Co., Ltd., Shanghai, China) before being used.

Drug pretreatment. The purified, healthy neutrophils were separated into four parts. Part I was incubated in the migration medium. Part II and III were pre-incubated with pravastatin (20 μM, Sigma, 81131-70-6 (anhydrous)) and metformin hydrochloride (1 mM, Sigma, 1115-70-4) to mimic the treatment of diabetes, respectively [33]. Part IV was pre-incubated with pravastatin and metformin hydrochloride mixture.

Glucose pretreatment. The purified, healthy neutrophils were separated into four parts and pretreated with 5, 25, 50, and 70 mM of d-glucose (G7021, Sigma, St. Louis, MO, USA).

AGEs pretreatment. The purified, healthy neutrophils were separated into four parts and pretreated with 0, 0.2, 0.5, and 1 μM of AGEs (2223-10, Wuhan Emeijie Technology Co., Ltd., Wuhan, China).

AGEs and FPS-ZM1 pretreatment. The purified, healthy neutrophils were separated into four parts and incubated in the migration medium, 0.2 μM of AGEs without the FPS-ZM1 (Haoxin Biotechnology Co., Ltd., Hangzhou, China), 0.2 μM of AGEs mixed with 0.2 μM of FPS-ZM1, and 0.2 μM of AGEs mixed with 0.5 μM of FPS-ZM1, respectively.

The various foregoing solutions were prepared using a migration medium. The incubation conditions were set as 5% of CO_2_, 95% of humidity, and 37 °C for 60 min. All pretreated neutrophils needed to be carefully washed and then resuspended in the new migration medium before experiments.

### 2.4. Cell Migration Experiments

#### 2.4.1. Cell Loading

The detailed information of the neutrophils and reagents distribution in the F4-chip for the following experiments are provided in Figures S3–S7. Before loading neutrophils, all of the F4-chip holes were emptied. The cells were then loaded from the cell loading inlets. The pressure difference between the inlets and outlets pushed the neutrophils to flow and be aligned beside the migration channels due to the cell docking structures.

#### 2.4.2. Gradient Generation

For screening the optimal fMLP concentration, the chemoattractant solutions (i.e., 0, 10, 100, 1000 nM of fMLP) were added to the F4-chip. For other experiments, 100 nM of fMLP gradient were generated in the four migration channels.

#### 2.4.3. Image Capture

The inverted fluorescence microscope recorded the migration progress of neutrophils with a temperature controller to set the ambient temperature to be 37 °C. The image capture frequency was set as 6 frames per min for 15 min.

### 2.5. Data Analysis

**Figure 2:**
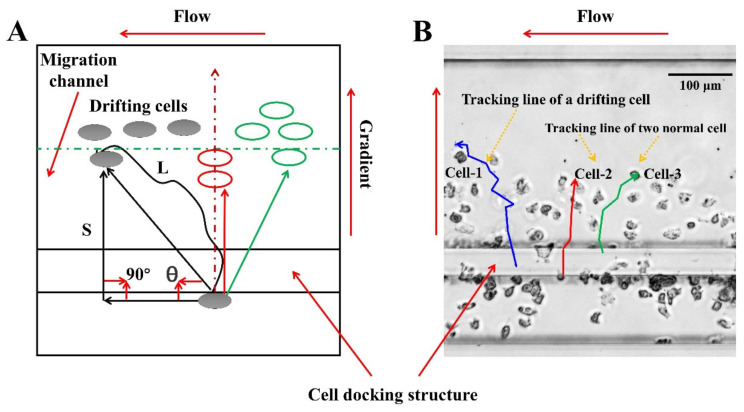
Cell migration analysis method. (**A**) Schematic diagram of the trajectory of drifting cells and normal cells. If θ is less than 90 degrees, the cells are migrating along with the flow. If θ is greater than 90 degrees, the cell migration progress is less affected by the flow. (**B**) The real trajectory of the drifting cells and normal cells in the cell migration channel.

FITC–dextran (Sigma-Aldrich, MO; 10 kDa; final concentration 5 μM in RPMI-1640) was firstly mixed with the chemoattractant solution by following the previously established method, and then loaded in the microfluidic chip to verify gradient by measuring the fluorescence intensity [31,32,35]. Gradient curves were analyzed using the software that comes from the microscope. For chemotactic analysis, the migration images were artificially tracked and analyzed using the ImageJ software (Version 1.8.0, National Institutes of Health, Bethesda, MD, USA). At least 30 cells were chosen and tracked in each channel. Abnormal drift and involuntary movement of neutrophils should be excluded when doing the tracking. The neutrophil chemotaxis was characterized by the drifting index (AI), the average cell velocity (V), the displacement along the gradient (S), and the whole movement distance (L). AI means the ratio of the cell number moving away from the gradient and toward the flow and all tracked cell numbers (Figure 2). The mathematical relationship between AI, V, S and L is shown in Equations (1)–(3), where T is the time interval of image acquisition. Especially, the angle between the S and the flow direction is defined to be 90°. Each experiment was repeated at least 3 times.

(1) $$AI=\frac{Drifting\;Cell\;number_{\left(\theta \;<\;90{}^{\circ}\right)}}{Total\;tracking\;cell\;number}$$

(2) $$V=\frac{L}{T}$$

(3) S=The displacement of a cell migraiton along gradient

## 3. Results

### 3.1. The F4-Chip and Gradient Characterization

**Figure 3:**
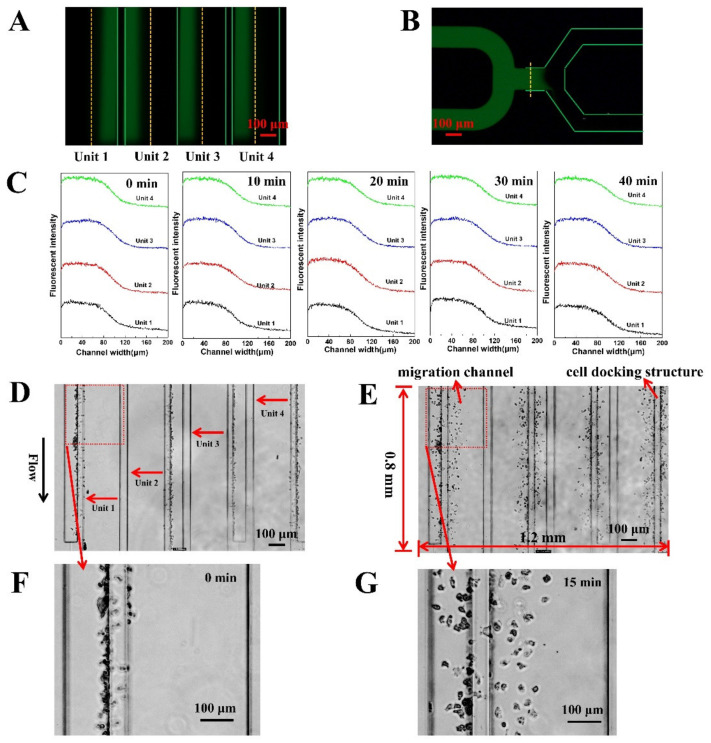
F4-chip characterization. (**A**) Fluorescent images in the four main channels; (**B**) Fluorescence image of the balance channel; (**C**) Gradient curves in unit 1, 2, 3, and 4 at 0 min, 10 min, 20 min, 30 min, 40 min; (**D**) Images of cell distribution beside cell docking structure; (**E**) Images of cell distribution in the migration channel after gradient application. Neutrophils need to deform and squeeze through the cell docking structure before migration into the migration channels; (**F**) An enlarged image for tracking analysis (0 min); (**G**) An enlarged image for tracking analysis (15 min).

The basic design of the F4-chip was referred to the published microfluidic devices from our and some other groups [31,34,39]. In this study, the F4 chip was equipped with four independent units (Figure 1A–C), which were very important for the cell migration experiments. Specifically, unit 1 (unit 4) and unit 2 (unit 3) have a similar structure and are arranged symmetrically, and so are unit 2 and unit 3. Sizes of units 1, 2, 3, 4 were optimized to fit into a single FOV under the objective lens of a microscope. Our group is considering further increases in the detection throughput of the F4-chip by more compact designs to allow more advanced experiments. The structure of the imaging area under an inverted fluorescence microscope is shown in Figure 1B. Each independent unit has its specific chemokine loading inlet, migration medium loading inlet, cell loading inlet, and outlet, thereby permitting four independent migration experiments by building different gradients or loading neutrophils pretreated with different drugs (Figure 1C). The F4-chip allowed rapid pumpless stable chemical gradient generation through laminar flow and convective diffusion mechanisms. For the F4-chip, the four gradient generations are very quick; thus, we usually first injected the migration medium into the different test units and then injected the chemokines at the fastest speed to minimize the impact of the time gap testing results. The gradient profiles in unit 1 (unit 4) and unit 2 (unit 3) were also simulated using COMSOL software (Version 5.5.0.359, COMSOL Group Inc., Stockholm, Sweden). We applied the 2D incompressible Navier–Stokes equation to simulate the flow field and the transient convection-diffusion equation to simulate the concentration distribution. The parameters of N-formylmethionine-leucyl-phenylalanine (fMLP, Sigma-Aldrich) were adapted from the literature [40]. The results showed that although there are structural differences between unit 1 (unit 4) and unit 2 (unit 3), the concentration gradients at the initial/termination positions of the four migration channels in the cell imaging area were consistent, which was essential for observing the cell migration behavior in four channels simultaneously (Figure S8). The gradient test results further confirmed that the pressure difference between the chemokine loading inlet and the migration medium loading inlet was offset at the balance channel (Figure 3A,B), which was beneficial for generating an identical gradient curve in the migration channel. Especially, the gradient can remain stable for at least 40 min which was enough for neutrophil chemotaxis experiments (Figure 3C). Moreover, the cell docking structure located next to the migration channel was confirmed to calibrate the original position of the neutrophils (Figure 1D and Figure 3D). Neutrophils can be initially calibrated as neatly as possible along the migration channel as their diameter is larger than the height of the cell docking structure (Figure 1D and Figure 3D). Upon creation of the chemoattractant gradient, neutrophils will deform and then transmigrate the cell docking structure to migrate into the migration channel (Figure 3E, Supporting Video S1). All neutrophils’ initial positions were aligned relative to the chemoattractant gradient, which was an advantage for improving the analyst accuracy. Furthermore, the transmigration of neutrophils through the docking structure partially mimicked the progress of the neutrophil transmigration through the vascular wall and movement into the tissues. When doing manual tracking, the microscopic image sequences were imported into the ImageJ software (Version 1.8.0, National Institutes of Health, Bethesda, MD, USA). Then a single channel was cut out and enlarged so that the cells of interest could be identified (Figure 3F,G).

### 3.2. On-Chip Neutrophil Chemotaxis Assay

**Figure 4:**
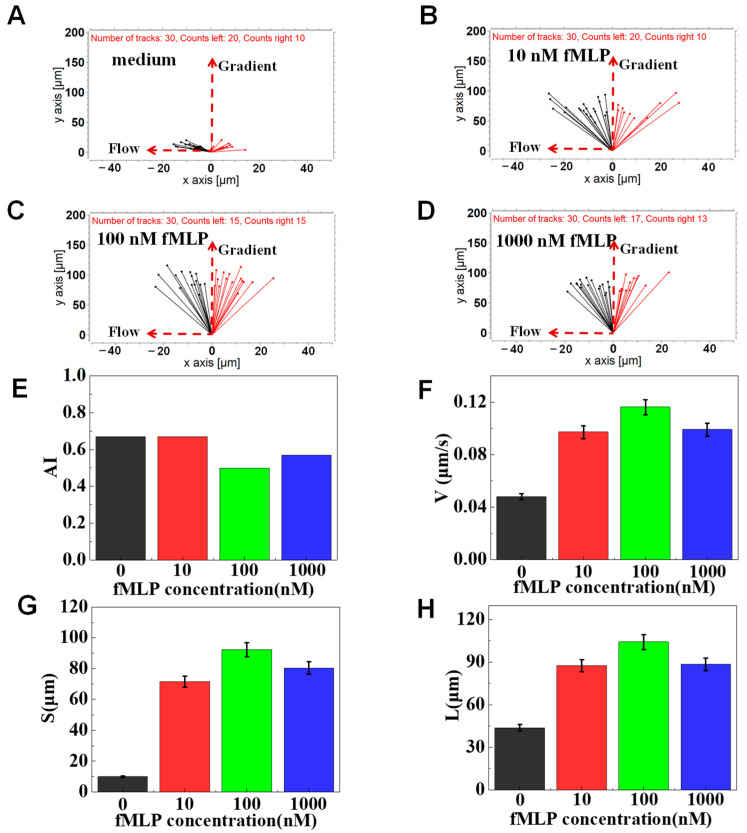
Chemotaxis of healthy cells to the fMLP chemoattractant. (**A**) The connecting line between the initial position and the end position of the cell in a migration medium. (**B**) The connecting line between the initial position and the end position of the cells in the 10 nM of fMLP gradient. (**C**) The connecting line between the initial position and the end position of the cells in the 100 nM of fMLP gradient. (**D**) The connecting line between the initial position and the end position of the cells in the 1000 nM of fMLP gradient. (**E**–**H**) Comparison of quantitative cells migration parameters including drifting index (AI), cell velocity (V), the migration displacement in the gradient direction (S), and the total migration distance (L).

fMLP has been demonstrated to induce neutrophil chemotaxis [23]. Thus, we first validated the function of F4-Chip by testing the neutrophil transmigration in the fMLP gradients (10, 100, and 1000 nM). The results of a representative experiment showed that the neutrophils strongly migrate towards the fMLP gradients (Figure 4A–D). The AI values for the 100 nM and 1000 nM groups are calculated to be lower than that for the 10 nM groups. Further, V, S, L of 10, 100, 1000 nM groups were verified to be higher than the control group (Figure 4E–H). However, the V, S, L in the 100 nM fMLP gradient were consistently the highest. A possible reason is that appropriate fMLP can bind to corresponding high-affinity receptor proteins on neutrophils, activate neutrophils, and cause chemotaxis. However, suppose the concentration of fMLP exceeds a certain threshold. In that case, it can bind to neutrophils’ corresponding low-affinity receptor proteins, leading to the release of harmful substances such as free radicals, lysosomal enzymes, and cytokines (such as IL-6, etc.), thereby affecting their chemotaxis [41,42]. In addition, this dose-related neutrophil chemotaxis towards the fMLP gradients was partly attributed to the cell docking structure of F4-Chip. If the cells were randomly seeded in the migration channel, the response of different cells to the chemokines will be different. We believe if the docking structure was integrated into the F4-chip, all neutrophils would have the same initial perception of the gradient so that the dose-related chemotaxis could be characterized by V, S, L. Thus, all of the following migration experiments were carried out by using 100 nM of fMLP gradient.

**Figure 5:**
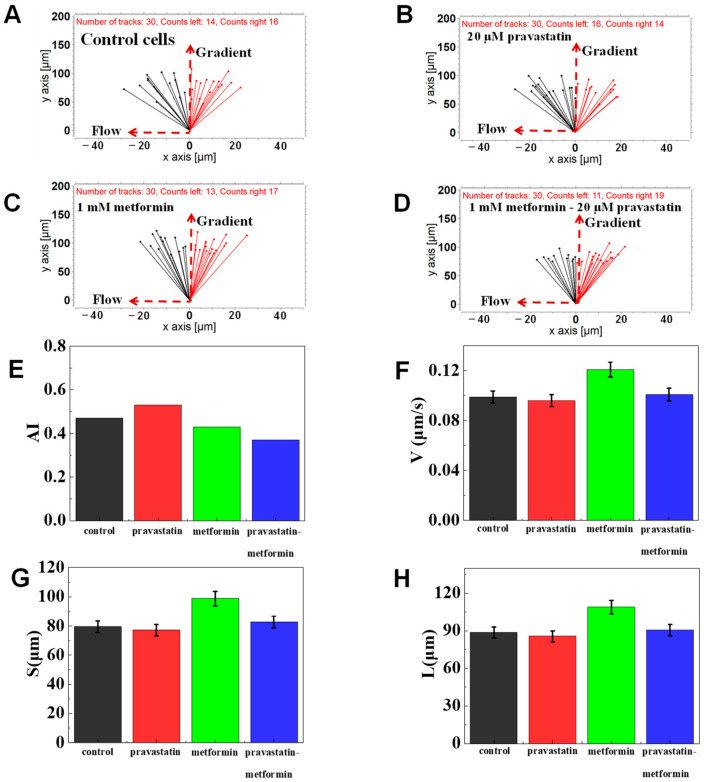
Chemotaxis of drugs-treated cells in 100 nM of fMLP chemoattractant. (**A**) The connecting line between the initial position and the end position of control cells. (**B**) The connecting line between the initial position and the end position of cells pretreated with 20 μM pravastatin. (**C**) The connecting line between the initial position and the end position of the cells pretreated with 1 mM metformin. (**D**) The connecting line between the initial position and the end position of the cells pretreated with 1 mM metformin and 20 μM pravastatin. (**E**–**H**) Comparison of quantitative cell migration parameters including drifting index (AI), cell velocity (V), the displacement along the gradient (S), and the whole movement distance (L).

Metformin and pravastatin have been demonstrated to change the chemotaxis and phagocytic ability of neutrophils [43,44]. We further validated the F4-Chip by studying the chemotaxis behaviors of healthy neutrophils exposed to metformin, pravastatin, and their mixture. The results of a representative experiment showed that pravastatin-treated cells showed stronger drifting along with the flow than the control cells (Figure 5A–D). In particular, the AI values of cells pretreated with migration medium, pravastatin, metformin, and their mixture were calculated to be 0.47, 0.53, 0.43, 0.37, respectively (Figure 5E). Further, the migration speed of metformin-treated cells was found to be higher (as measured by V) than that of the control, pravastatin-, mixture-treated group (Figure 5F–H). This result was similar to previous studies reporting the suppressed chemotaxis of neutrophils pretreated with pravastatin [44] and enhanced chemotaxis speed of neutrophils pretreated with metformin [43]. Considering these results, F4-chip was successfully demonstrated by testing the chemotaxis of normal neutrophils, neutrophils pretreated by antidiabetic and lipid-lowering drugs exposed to fMLP gradients with satisfying fidelity.

### 3.3. The Function of Glucose on Neutrophil Chemotaxis

**Figure 6:**
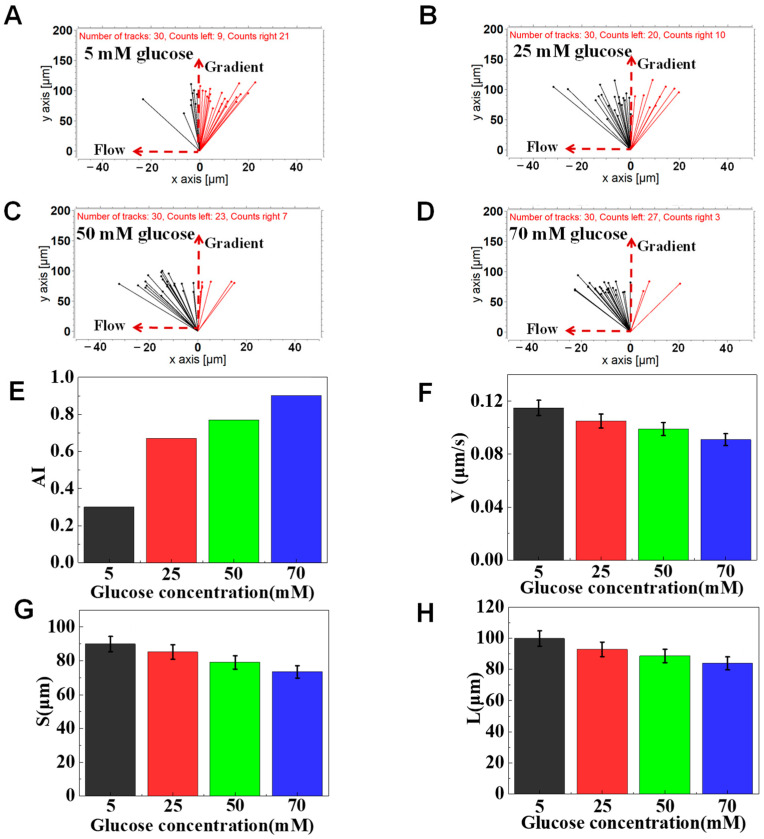
Chemotaxis of glucose-treated cells in 100 nM of fMLP gradient. (**A**) The connecting line between the initial position and the end position of cells pretreated with 5 mM glucose. (**B**) The connecting line between the initial position and the end position of cells pretreated with 25 mM glucose. (**C**) The connecting line between the initial position and the end position of cells pretreated with 50 mM glucose. (**D**) The connecting line between the initial position and the end position of cells pretreated with 70 mM glucose. (**E**–**H**) Comparison of quantitative cell migration parameters including drifting index (AI), cell velocity (V), the displacement along the gradient (S), and the whole movement distance (L).

To evaluate the possible impact of a high concentration of glucose on the chemotaxis of neutrophils, mimicked neutrophil transmigration was tested using the F4-Chip. Usually, the normal fasting blood glucose value is between 4 and 6 mM. Thus, 5 mM of glucose was set as the control group in the present study, and 25, 50, and 70 mM of glucose were set as experimental groups. The results of a representative experiment indicated that different concentrations of glucose pretreated cells could effectively transmigrate through the cell docking structure and migrate along the fMLP gradient in the migration channel. Interestingly, we found a stronger drifting of 25, 50, and 70 mM glucose pretreated neutrophils along with the flow than that of the control group, implying the weakened chemotaxis of neutrophils towards a 100 nM fMLP gradient (Figure 6A–D). As measured by AI, we found that different concentrations of glucose pretreatment led to the quantitative differentiation on neutrophil chemotaxis (Figure 6E). The result was the same as the previous studies that found high concentrations of glucose could induce the change of neutrophil adhesion and limited neutrophil recruiting toward infected tissues [11,12]. Further, the migration speed calculated was found to be decreased as the glucose concentration increased (as measured by V) (Figure 6F). In specific, the average migration speed of control group was 0.115 μm/s, which was higher than that of 25 mM group (0.105 μm/s), 50 mM group (0.099 μm/s), and 70 mM group (0.091 μm/s). Gradient direction displacement (as measured by S) and total migration distance (as measured by L) analysis also showed the gradually increased inhibition in the chemotaxis of high glucose-exposed cells comparing to the control group (Figure 6G,H). In short, high concentrations of glucose treatment can induce weakened chemotaxis of transmigrated neutrophils than the control cells. The foregoing results were consistent with previously reported results of high concentrations of glucose inhibiting the chemotaxis of neutrophils [11].

### 3.4. The Function of AGEs on Neutrophil Chemotaxis

**Figure 7:**
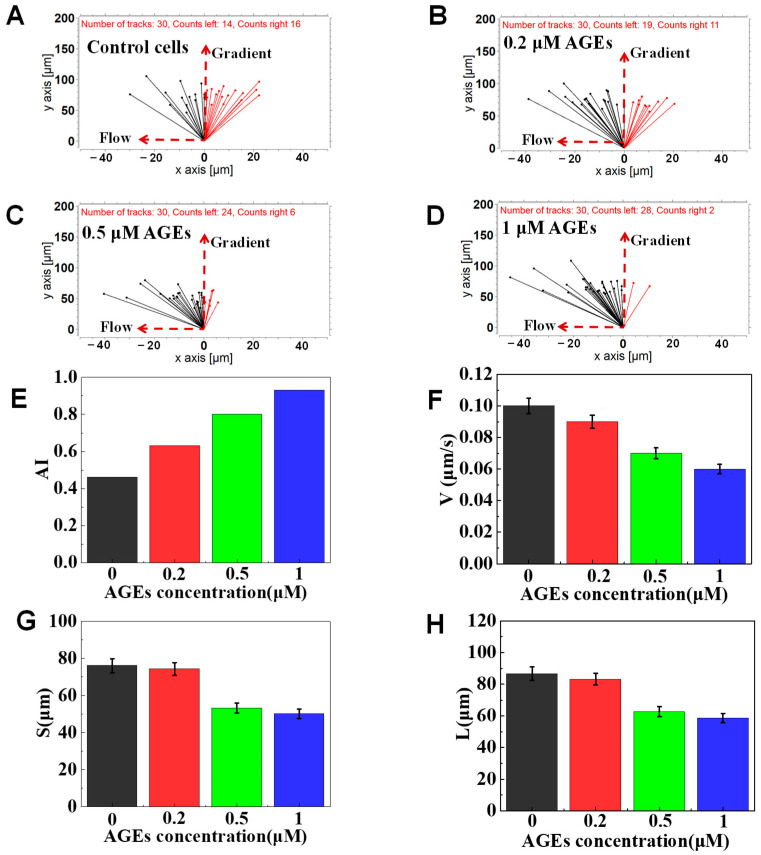
Chemotaxis of AGEs-treated cells in 100 nM of fMLP gradient. (**A**) The connecting line between the initial position and the end position of cells pretreated with 0 μM AGEs. (**B**) The connecting line between the initial position and the end position of cells pretreated with 0.2 μM AGEs. (**C**) The connecting line between the initial position and the end position of cells pre-treated with 0.5 μM AGEs. (**D**) The connecting line between the initial position and the end position of cells pretreated with 1 μM AGEs. (**E**–**H**) Comparison of quantitative cell migration parameters including drifting index (AI), cell velocity (V), the displacement along the gradient (S), and the whole movement distance (L).

The normal serum AGEs levels in normal patients (non-diabetic, normal renal function) were around 0.13 μM. AGEs levels were elevated more than 2-fold in diabetic patients and about 8-fold in diabetics with hemodialysis [19,45]. Thus, here we focused on the effect of high concentrations of AGEs on the chemotaxis phenotyping of neutrophils. In a representative experiment, we found that different concentrations of AGEs pretreated cells could effectively transmigrate through the docking structure and migrate in the migration channels in different ways (e.g., different speeds and migration distances). In particular, AGEs pretreated cells showed stronger drifting along with the flow than the control cells (Figure 7A–D). In addition, the AI values of cells pretreated with 0, 0.2, 0.5, and 1.0 μM of AGEs were calculated to be 0.46, 0.63, 0.8, 0.93, respectively (Figure 7E), implying the weakened neutrophil chemotaxis like the glucose pretreated neutrophils. Further, we found that the AGEs inhibition of neutrophil migration ability was dose-dependent (as measured by V, S, L). The V of the control cells was 0.1 μm/s, higher than that of cells pretreated with 0.2 (0.09 μm/s), 0.5 (0.07 μm/s) and 1 (0.06 μm/s) μM AGEs (Figure 7F). Gradient direction displacement and total migration distance also showed the gradually increased inhibition in chemotaxis (Figure 7G,H). The results further confirmed that AGEs treatment could induce weakened chemotaxis of transmigrated neutrophils than the control cells in 100 nM of fMLP gradient.

### 3.5. The Function of FPS-ZM1 on Neutrophil Chemotaxis

**Figure 8:**
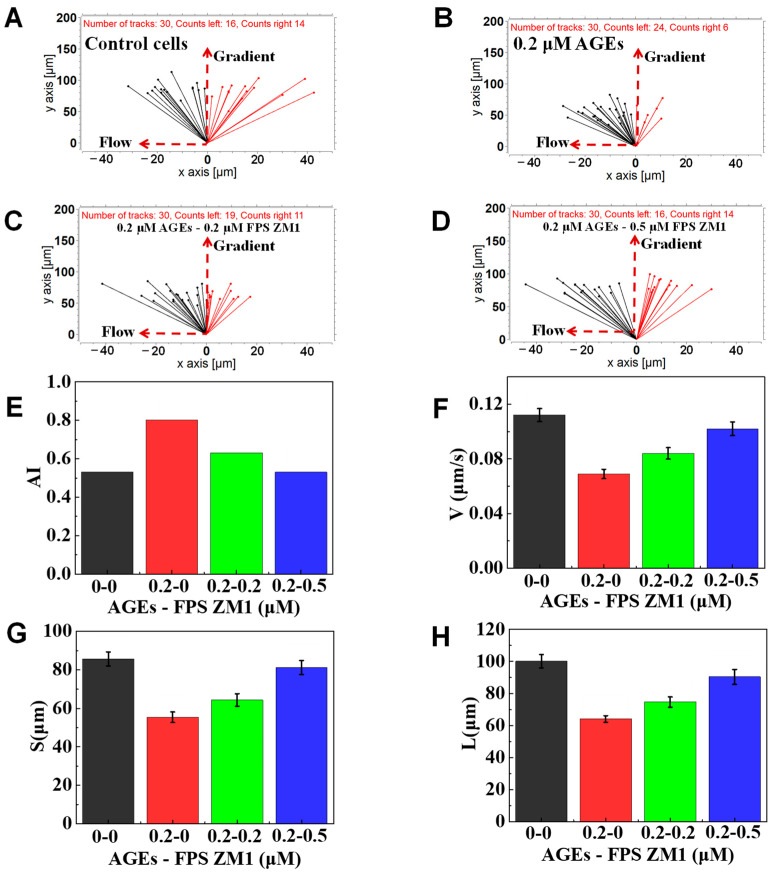
Chemotaxis of AGEs, FPS ZM1, and their mixture-treated test cells in 100 nM of fMLP gradient. (**A**) The connecting line between the initial position and the end position of normal cells. (**B**) The connecting line between the initial and end position of cells pretreated with 0.2 μM AGEs and 0 μM FPS ZM1. (**C**) The connecting line between the initial and end position of cells pretreated with 0.2 μM AGEs and 0.2 μM FPS ZM1. (**D**) The connecting line between the initial and end position of cells pretreated with 0.2 μM AGEs and 0.5 μM FPS ZM1 pretreated cells. (**E**–**H**) Comparison of quantitative cell migration parameters including drifting index (AI), cell velocity (V), the displacement along the gradient (S), and the whole movement distance (L).

Schuetz P and Advani et al. found that when AGEs activated the neutrophils, intracellular Ca^2+^ signal transduction was abnormal, increasing intracellular Ca^2+^, thus inhibiting their chemotaxis [9,46]. Recently, Manli Na et al. found that if the RAGE was blocked, neutrophils’ rolling, adhesion and migration ability were restored [47]. To further understand whether the inhibition effects of AGE on the chemotaxis of neutrophils could be relieved, we added a RAGE inhibitor (FPS-ZM1) to the incubation reagents with AGEs to further explore the basic mechanism of AGEs inhibiting the chemotaxis of neutrophils. The results of a representative experiment confirmed again that 0.2 μM AGEs treatment could weaken the chemotaxis of transmigrated neutrophils (a stronger drifting along with the flow) than the control cells (Figure 8A,B). Compared with the 0.2 μM AGEs group, 0.2 μM AGEs + 0.2 μM FPS-ZM1 treatment can relieve the effect of AGEs to the chemotaxis of neutrophils, expressing a partly enhanced ability to migrate toward the gradient (Figure 8B,C). In addition, we found that the 0.2 μM AGEs + 0.5 μM FPS-ZM1 treatment group could further suppress the effect of AGEs on the chemotaxis of neutrophils compared with the 0.2 μM AGEs + 0.2 μM FPS-ZM1 treatment group, showing as fully restored chemotaxis ability (Figure 8C,D). Specifically, the calculated AI in 0.2 μM of the AGEs group was higher than the control group, indicating the effect of AGEs on the chemotaxis of neutrophils (Figure 8E). In addition, the calculated AI gradually was decreased from 0.8 to 0.53 as the dose of FPS-ZM1 increased from 0.2 to 0.5 μM, indicating that FPS-ZM1 relieved the effect of AGEs on the chemotaxis of neutrophils. Further, the V of the control cells was calculated to be 0.112 μm/s, higher than 0.2 μM AGEs (0.069 μm/s), and the V was gradually increased from 0.084 μm/s to 0.102 μm/s as the dose of FPS-ZM1 increased (Figure 8F). Gradient direction displacement and total migration distance analysis also showed the increased recovery in chemotactic migration ability as the dose increases of FPS-ZM1 (Figure 8G,H). In summary, the above experimental results showed that the FPS-ZM1 mixed with AGEs could hinder the binding between AGEs and RAGE located on the neutrophil, thereby keeping the normal chemotaxis of neutrophils.

## 4. Discussion

We, in this paper, designed a novel F4 chip that can carry out four independent chemotaxis experiments simultaneously. The F4-chip included four cell docking structures to align the initial position of cells beside the migration channels before adding chemokine. The initial movement position of cells can be confined by a mechanical structure of the cell docking area instead of firming the cells to the substrate by adhesion, which is important for result analysis.

We first validated the F4-chip using the healthy neutrophils exposed to the different concentrations of fMLP and confirmed that all of the following experiments in the current research would be carried out using a 100 nM fMLP gradient. Then, we further validated the feasibility of F4-chip for clinically relevant tests by using healthy neutrophils pretreated with common diabetic drugs (i.e., metformin and pravastatin). Interestingly, we observed the suppressed neutrophil chemotaxis treated with pravastatin and enhanced neutrophil chemotaxis speed with metformin. The chemotaxis upregulation of neutrophils can be due to metformin-mediated 5′ adenosine monophosphate-activated protein kinase activation [48], which can help improve leukocyte oxidative stress and impaired neutrophil functions to a healthy point in diabetics [49].

Diabetes mellitus is a chronic metabolic disorder characterized by a hyperglycemic condition and often accompanied by various complications, such as neuropathy, nephropathy, and retinopathy [50]. Hyperglycemia is a key abnormality of diabetes and plays a key role in inflammation development of diabetes and related complications. A series of studies have proven that hyperglycemia could reduce migration [51], proliferation [52], collagen synthesis [53], and increase apoptosis [54] in various cell types. Specifically, the high concentration of glucose has been confirmed to interfere with metabolites and enzymes related to the physiological functions of neutrophils [6]. We this research demonstrated the general weakening effect of glucose on the chemotaxis of neutrophils by using the F4-chip. Consistently, the weakened chemotaxis was accompanied by a drift of cells along the flow direction, suggesting that adhesion on the glass substrate of the glucose pretreated neutrophils was possibly altered. This may be due to when neutrophils are in a high concentration of glucose solution, the biological activity of neutrophils is affected due to osmotic pressure and other factors, so that the migration ability of neutrophils is greatly affected.

In addition, it has been confirmed that chronic hyperglycemia will result in the generation of AGEs. The formation of AGEs is due to a non-enzymatic reaction between the glucose and amino acids of proteins. The increasing accumulation of AGEs in the tissues and serum of diabetic patients is closely related to the pathogenesis of vascular complications [55]. Many cell receptors for AGEs have been discovered, the most typical of which is RAGE, a member of the immunoglobulin receptor superfamily [56,57]. Researchers have demonstrated the impaired transendothelial migration of AGEs pretreated neutrophils in fMLP gradient generated in the transwell chamber, suggesting that AGEs might also reduce the ability of neutrophils to respond to the physiological chemotactic stimuli [19]. We used the F4-chip and confirmed the impaired chemotaxis of neutrophils pretreated with AGEs. In particular, as the concentration of AGEs increases, neutrophils move away from the gradient direction (toward the direction of flow), and their migration speed gradually decreases, which partly demonstrated the previously proposed mechanism which thought that AGEs induced the aberrant signal processing and altered neutrophil chemotaxis [10]. Furthermore, our results showed that the FPS-ZM1 could hinder the binding of neutrophil RAGE to AGEs and block the effect of AGEs on neutrophil chemotaxis.

## 5. Conclusions

In short, we here provided a new F4 chip. We successfully studied the effect of high concentrations of glucose and AGEs on the neutrophil chemotaxis, which suggested that patients with diabetes should manage serum AGEs and pay attention to the blood glucose indexes. In the following, we will further collect the diabetes patients’ blood samples and lay the foundation for clinical diagnostic, treatment applications, and prognostic monitoring.

## Data Availability

Data is contained within the article or supplementary material.

## Supplementary Materials

The following are available online at https://www.mdpi.com/article/10.3390/mi12080944/s1, Figure S1: The preparation progresses of the F4-chip. Figure S2: The detailed neutrophil pretreatment method for various experiments. Figure S3: Experiment setup method of neutrophil chemotaxis to known fMLP chemoattractant in the F4-Chip. Figure S4: Experiment setup method of testing the function of pravastatin and metformin on neutrophil chemotaxis in the F4-Chip. Figure S5: Experiment setup method of testing the function of glucose on neutrophil chemotaxis in the F4-Chip. Figure S6: Experiment setup method of testing the function of AGEs on neutrophil chemotaxis in the F4-Chip. Figure S7: Experiment setup method of testing the function of FPS-ZM1 on neutrophil chemotaxis in the F4-Chip. Figure S8: Gradient simulation results of microfluidic chip. Video S1: Upon creation of the chemoattractant gradient, neutrophils will deform and then transmigrate the cell docking structure to migrate into the migration channel.
