# Supplementary material for: A Novel Microfluidic Device for the Neutrophil Functional Phenotype Analysis: Effects of Glucose and Its Derivatives AGEs
Source: Micromachines (Basel). 2021 Aug 11;12(8):944. doi: 10.3390/mi12080944 (PMC8399494; doi:10.3390/mi12080944)
Supplement: Supplementary file 1 [file micromachines-12-00944-s001.zip › Supplementary Material - Figure.pdf]

A novel microfluidic device for the neutrophil functional phenotype analysis: Effects of glucose and its derivatives AGEs

Ke Yang[*a], Xiao Yang[b], Chaoru Gao[b], Changyi Hua[a], Chenggang Hong[c], and Ling Zhu[a*]

a. Anhui Institute of Optics and Fine Mechanics, Hefei Institutes of Physical Science, Chinese Academy of Sciences, Hefei 230031, China. E-mail addresses: keyang@aiofm.ac.cn (Ke Yang), zhul@aiofm.ac.cn (Ling Zhu)

b. School of Biomedical Engineering, Anhui Medical University, Hefei 230032, China.

c. Hefei Zhongke Yikangda Biomedical Co., Ltd., Hefei 230088, China.

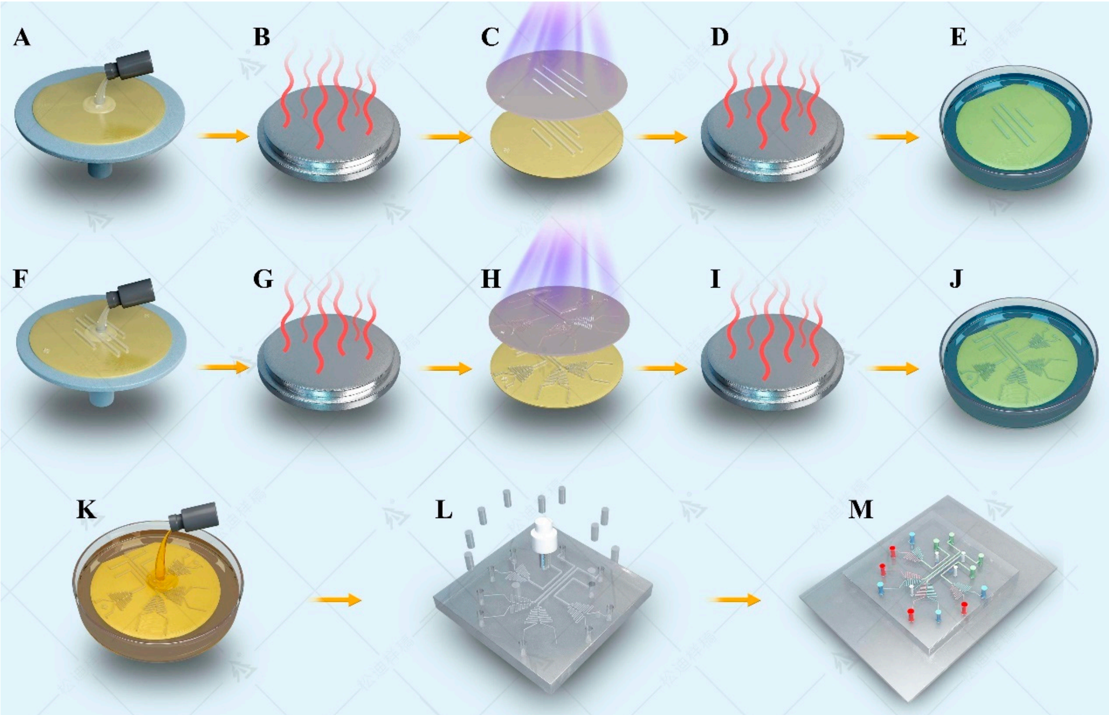

Figure S1. The preparation progresses of the F4-chip. (A) Pouring and spinning SU-8 (2 μm); (B) Baking at 95 ° C for 30 s; (C) UV exposure for 3 s; (D) Baking at 95 ° C for 30 s; (E) Developing for 30 s; (F) Pouring and spinning SU-8 (70 μm); (G) Baking at 65 ° C for 2 min, 95 ° C for 7 min; (H) UV exposure for 20 s; (I) Baking at 65 ° C for 2 min, 95 ° C for 7 min; (J) Developing for 5 min; (K) Pouring the PDMS; (L) Peeling and punching; (M) Bonding PDMS with glass slider.

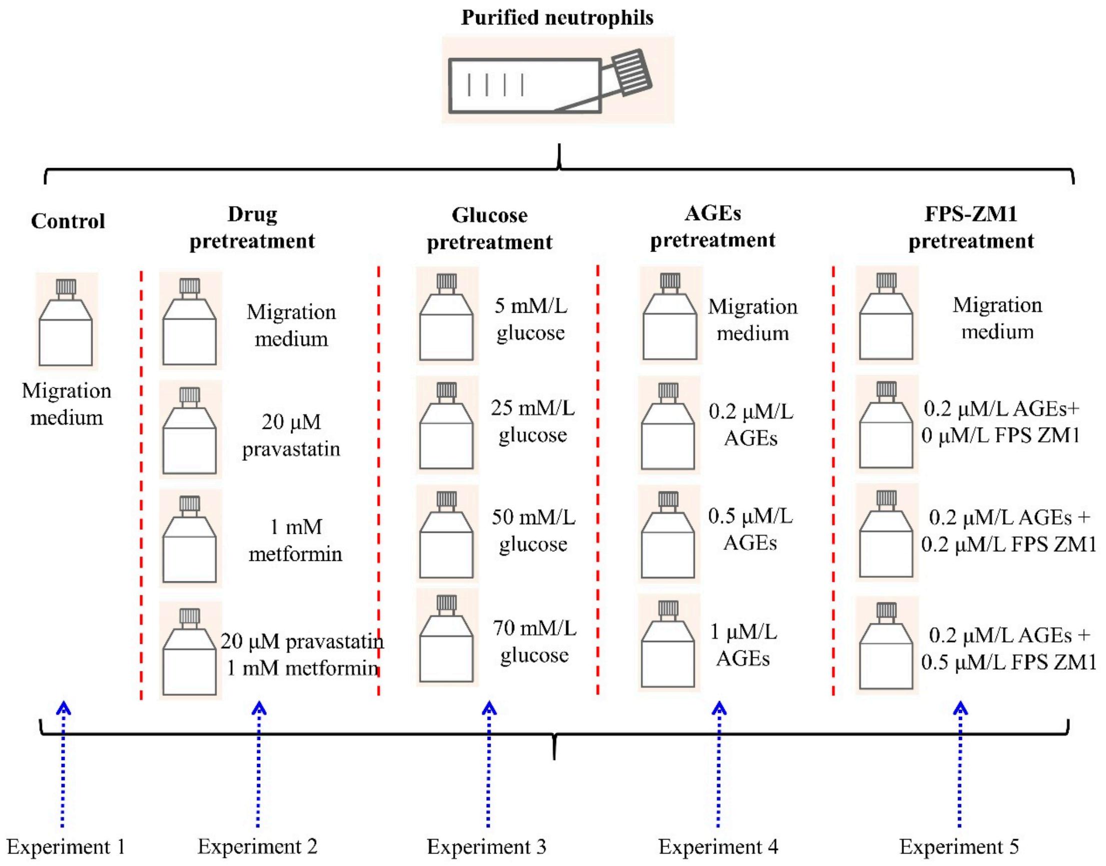

Figure S2. The detailed neutrophil pretreatment method for various experiments. Experiment 1: testing the chemotaxis of neutrophil to *f*MLP chemoattractant. Experiment 2: testing the function of metformin and pravastatin on neutrophil chemotaxis. Experiment 3: testing the function of glucose on neutrophil chemotaxis. Experiment 4: testing the function of AGEs on neutrophil chemotaxis. Experiment 5: testing the function of FPS-ZM1 on neutrophil chemotaxis.

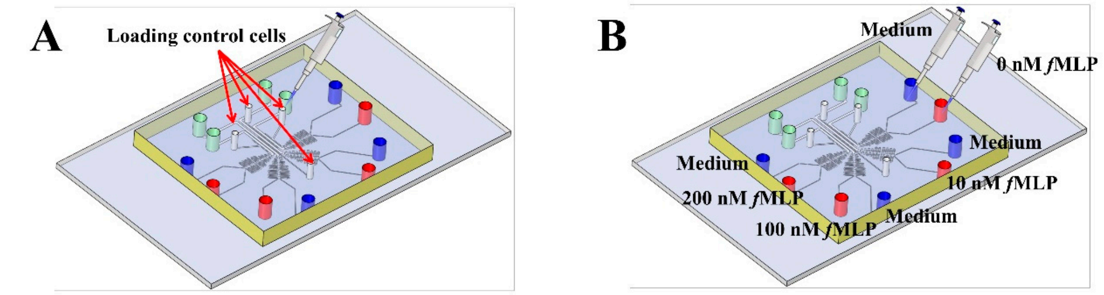

Figure S3. Experiment setup method of neutrophil chemotaxis to known *f*MLP chemoattractant in the F4-Chip.

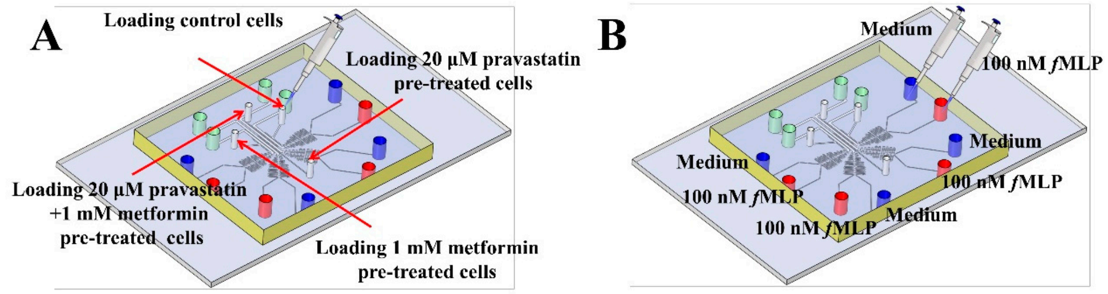

Figure S4. Experiment setup method of testing the function of pravastatin and metformin on neutrophil chemotaxis in the F4-Chip.

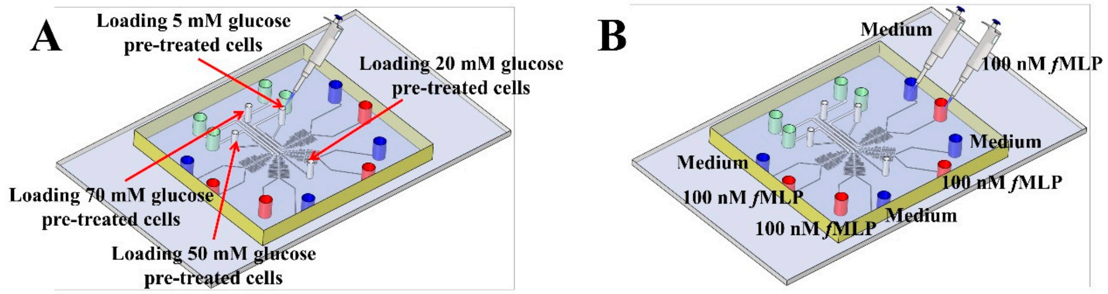

Figure S5. Experiment setup method of testing the function of glucose on neutrophil chemotaxis in the F4-Chip.

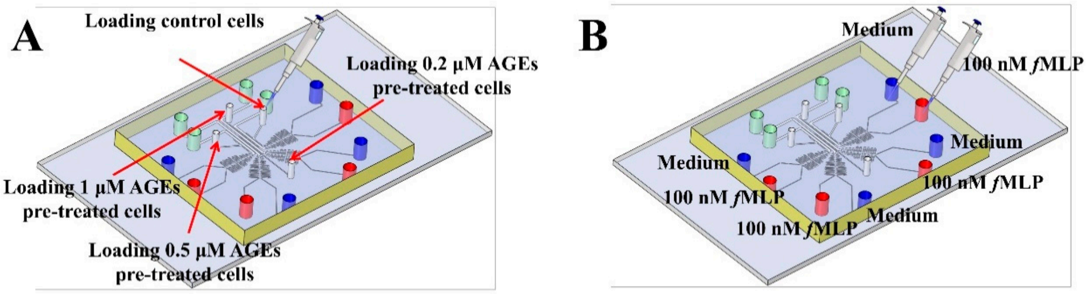

Figure S6. Experiment setup method of testing the function of AGEs on neutrophil chemotaxis in the F4-Chip.

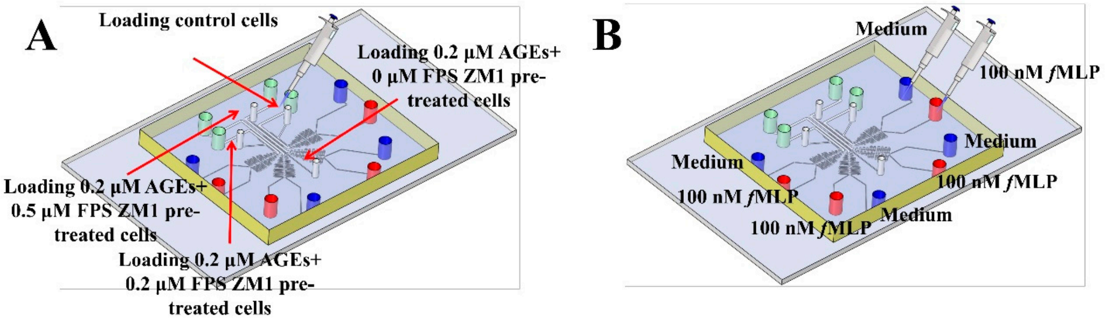

Figure S7. Experiment setup method of testing the function of FPS-ZM1 on neutrophil chemotaxis in the F4-Chip.

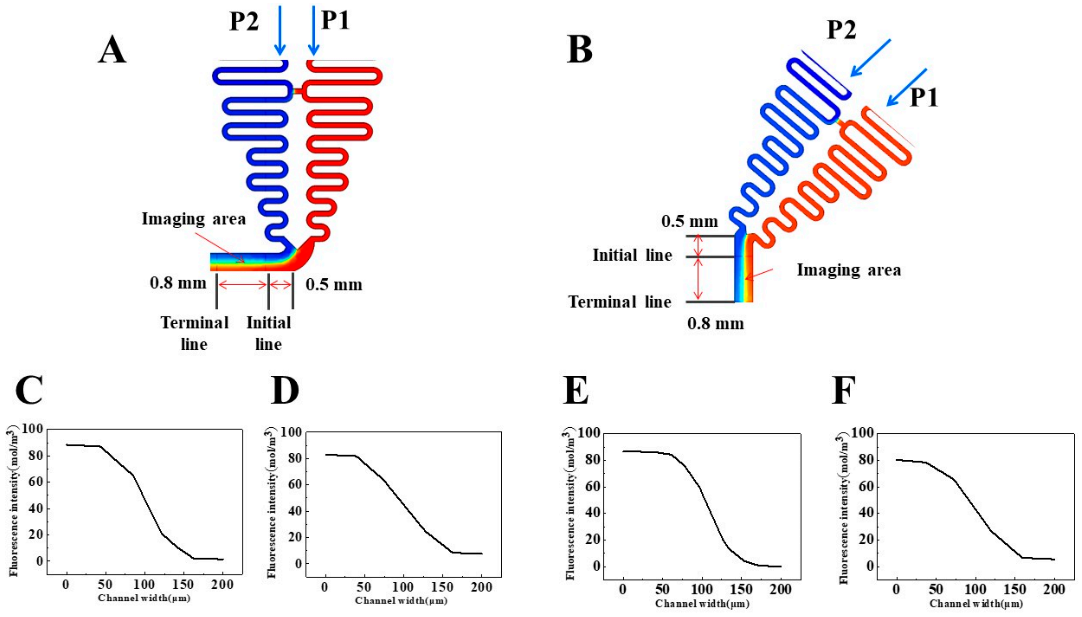

Figure S8. Gradient simulation results of microfluidic chip. (A) Gradient simulation image of unit 1 (unit 4); (B) Gradient simulation image of unit 2 (unit 3); (C) The gradient simulation curve at the initial line of the imaging area in unit 1 (unit 4); (D) The gradient simulation curve at the terminal line of the imaging area in unit 1 (unit 4); (E) The gradient simulation curve at the initial line of the imaging area in unit 2 (unit 3); (F) The gradient simulation curve at the terminal line of the imaging area in unit 2 (unit 3).
